# Supplementary material for: Structural variants in the barley gene pool: precision and sensitivity to detect them using short-read sequencing and their association with gene expression and phenotypic variation
Source: Theor Appl Genet. 2022 Aug 27;135(10):3511–29. doi: 10.1007/s00122-022-04197-7 (PMC9519679; doi:10.1007/s00122-022-04197-7)
Supplement: Supplementary file 1 — (pdf 3763 KB) [file 122_2022_4197_MOESM1_ESM.pdf]

SUPPLEMENTARY INFORMATION

Table S1: Sensitivity/precision of structural variant (SV) callers and combinations of them (for details see Material & Methods) to identify small insertions and deletions (2 - 49bp, INDELs) and translocations (50bp - 1Mb).

SV caller	Deletions (2 - 49bp)	Insertions (2 - 49bp)	Translocations (50bp - 1Mb)
Delly			85.6/76.0
Manta			89.4/100.0
Lumpy			83.2/82.4
GRIDSS	68.0/99.3	64.6/98.9	87.2/100.0
Pindel	92.4/97.9	87.5/98.7	
GATK	92.3/97.6	94.6/98.7	
Combination	95.5/98.9	94.8/98.7	95.4/99.8

Table S2: Predicted structural variants (SV) for PCR validation. Listed are all SV that were PCR validated including the names, sizes, primer positions, and the expected amplicon sizes. All sizes are given in bp.

	Primer position relative to SV start				Expected amplicon size	
SV names	left	right	2nd right	SV size	Morex	Unumli-Arpa
Del_A_1	-263	321		57	584	527
Del_A_2	-158	293		64	451	387
Del_A_3	-110	324		53	434	381
Del_A_4	-229	424		124	653	529
Del_A_5	-216	265		55	481	426
Del_A_6	-277	155		59	432	373
Ins_A_1	-167	243		57	353	410
Ins_A_2	-238	191		76	353	429
Ins_A_3	-234	258		91	401	492
Ins_A_4	-288	126		52	362	414
Ins_A_5	-266	239		57	448	505
Del_B_1	-391	2,704		1,937	3,095	1,158
Del_B_2	-462	4,446		4,144	4,908	764
Del_B_3	-374	3,687		2,940	4,061	1,121
Del_C_1	-364	316	11,313	10,778	680	899
Del_C_2	-103	280	5,692	5,355	383	440
Del_C_3	-231	375	28,406	27,937	606	700
Del_D_1	-262	120	287,036	286,558	382	740
Del_D_2	-361	371	91,956	91,411	732	906
Del_D_3	-248	224	54,918	54,481	472	685
Del_E_1	-169	348	460,621	460,240	517	550
Del_E_2	-279	239	405,578	405,029	518	828

Table S3: Proportion (%) of SV length categories for deletions, duplications, inversions, and insertions.

SV length category	Deletions	Duplications	Inversions	Insertions
A (50 - 300bp)	41.7	16.2	20.1	48.4
B (0.3 - 5kb)	30.3	21.7	16.5	5.7[1]
C (5 - 50kb)	26.4	55.9	25.9	
D (50 - 250kb)	1.5	5.5	24.4	
E (0.25 - 1Mb)	0.1	0.7	13.1	

[1]0.3 - 1kb; no insertion length detected for 45.9%

Table S4: Percentage of structural variant (SV) clusters or their closest neighboring single nucleotide variant (SNV) that show a maximum linkage disequilibrium (LD) estimate r^2_{max} to all SNV 1kb up and downstream of it. LD was calculated for three categories of minor allele frequencies (MAF) for SV clusters and the corresponding closest SNV.

Proportion (%) of r^2_{max}	r^2	MAF [0,0.2)	MAF [0.2,0.4)	MAF [0.4,0.5)	MAF [0,0.2)	MAF [0.2,0.4)	MAF [0.4,0.5)
		Between SV cluster and SNV			Between closest SNV to SV cluster and SNV		
Deletions	[1.0,0.8]	0.00	0.65	60.84	9.58	9.63	9.81
	(0.8,0.6]	54.70	73.65	13.08	79.98	79.86	79.64
	(0.6,0.4]	10.40	11.10	12.72	9.84	9.94	9.98
	(0.4,0.2]	27.67	8.32	7.62	0.00	0.00	0.00
	(0.2,0]	6.82	6.28	5.74	0.00	0.00	0.00
Insertions	[1.0,0.8]	0.00	0.56	60.70	9.53	9.67	9.85
	(0.8,0.6]	42.84	68.38	12.41	80.32	80.08	79.58
	(0.6,0.4]	11.57	11.79	12.37	9.58	9.70	9.95
	(0.4,0.2]	35.82	10.08	8.05	0.00	0.00	0.00
	(0.2,0]	9.48	9.19	6.46	0.00	0.00	0.00
Duplications	[1.0,0.8]	0.00	1.13	54.85	9.51	9.55	9.72
	(0.8,0.6]	33.66	66.22	13.40	80.28	80.12	79.85
	(0.6,0.4]	11.39	12.96	14.66	9.64	9.76	9.79
	(0.4,0.2]	44.93	10.67	9.78	0.00	0.00	0.00
	(0.2,0]	9.92	9.02	7.31	0.00	0.00	0.00
Inversions	[1.0,0.8]	0.00	0.96	50.00	10.11	9.29	9.94
	(0.8,0.6]	34.93	66.19	13.51	79.35	80.40	79.27
	(0.6,0.4]	11.56	13.60	15.55	9.98	9.69	10.16
	(0.4,0.2]	45.38	11.14	11.81	0.00	0.00	0.00
	(0.2,0]	7.96	8.09	9.11	0.00	0.00	0.00

Table S5: The optimal weights of the three predictors single nucleotide variants (SNV) and Indel (SNV&Indel), structural variants (SV) and gene expression that resulted in the highest prediction abilities for the seven traits heading time (HT), leaf angle (LA), plant height (PH), seed area (SA), seed length (SL), seed width (SW), and thousand grain weight (TGW).

Traits	SNV&INDELs	SV clusters	Gene expression	Prediction ability
HT	0.0	0.1	0.9	0.63
LA	0.0	0.4	0.6	0.79
PH	0.0	0.1	0.9	0.54
SA	0.9	0.0	0.1	0.74
SL	0.6	0.0	0.4	0.70
SW	0.0	1.0	0.0	0.75
TGW	1.0	0.0	0.0	0.86
Mean (median)	0.36 (0)	0.23 (0.1)	0.41 (0.4)	

Table S6: Inbred lines included in this study, their country of origin (CoO), row type, and year of release.

					Genome sequencing coverage		
Inbred name	BCC code	CoO	Row type	Year of release	seq	seq-trimmed	mapped
HOR1842	HOR1842	AFG	6	1935	27.4	26.3	25.9
HOR383	BCC1561	BGR	6	unknown	24.8	23.8	22.4
Sanalta	BCC929	CAN	2	1930	27.5	26.3	25.5
ItuNative	BCC502	CHN	6	unknown	23.6	22.7	21.3
Sissy	BCC1413	GER	2	1990	24.0	23.1	22.7
Georgie	BCC1381	GBR	2	1975	25.1	24.1	23.7
SprattArcher	BCC1415	GBR	2	1943	23.1	22.4	22.2
Lakhan	BCC533	IND	6	unknown	21.6	20.8	20.1
Kharsila	HOR11403	IND	6	before 1911	26.7	25.6	24.2
W23829/803911	HOR11374	ISR	2	unknown	23.6	22.7	22.4
Namhaebori	BCC667	KOR	6	unknown	22.3	21.6	20.4
IG128216	BCC118	LBY	6	1983	21.2	20.8	19.3
IG128104	BCC173	PAK	6	1974	23.8	22.9	22.4
K10693	BCC1491	RUS	6	unknown	21.0	20.2	19.8
IG31424	BCC190	SYR	2	1981	23.5	22.5	21.9
HOR12830	HOR12830	SYR	6	unknown	25.8	24.7	23.4
HOR7985	HOR7985	TUR	2	before 1969	23.3	22.3	22.3
K10877	BCC1503	TKM	6	unknown	25.5	24.4	23.7
HOR8160	HOR8160	TUR	2	before 1969	24.4	23.5	23.0
Ancap2	BCC807	URY	6	1950	27.0	25.9	24.6
CM67	BCC846	USA	6	1983	23.8	22.9	22.3
Kombyne	BCC893	USA	6	1975	21.5	20.5	19.9
Unumli-Arpa	BCC1470	UZB	2	unknown	23.5	22.6	22.1

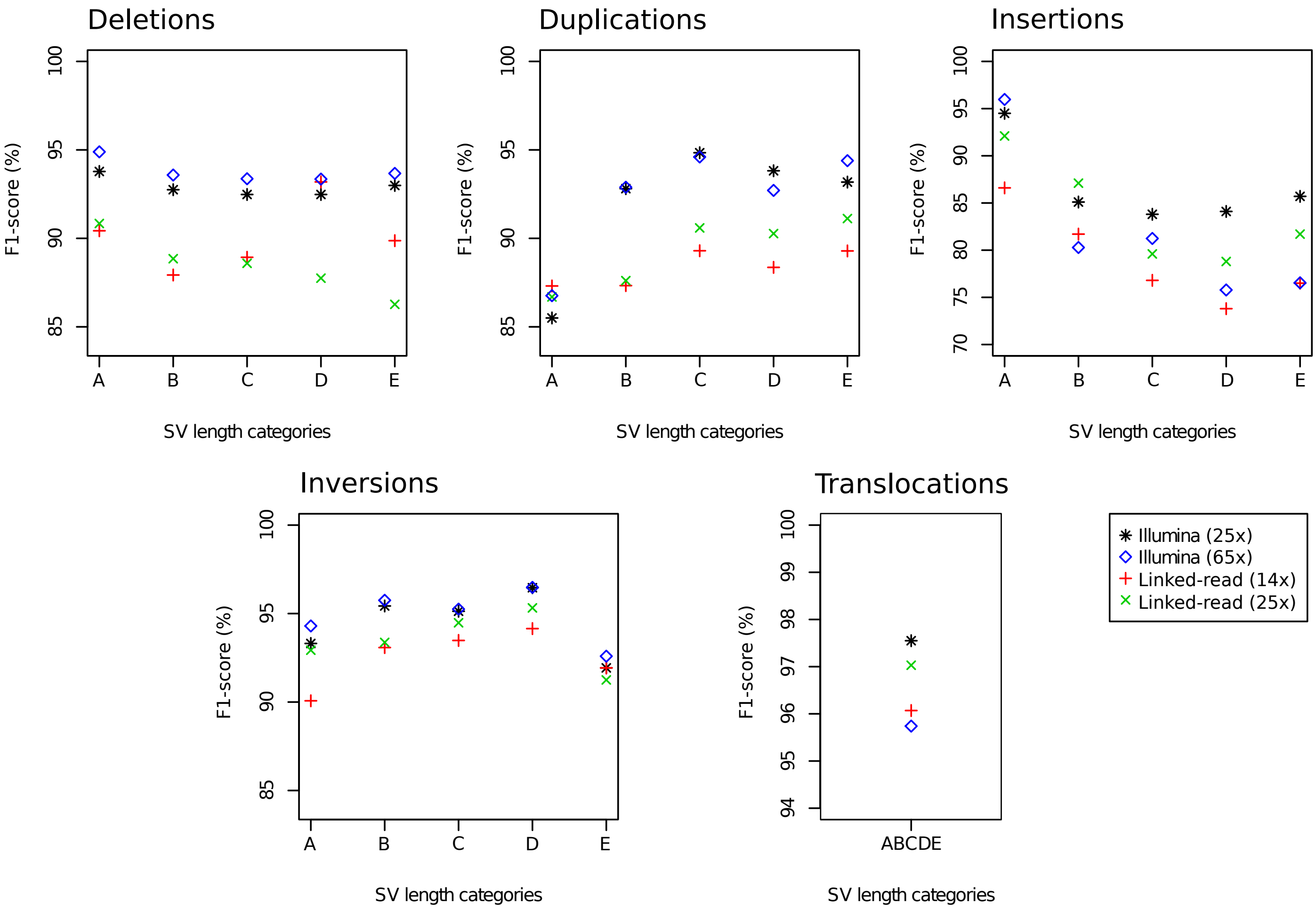

Fig. S1: F1-score, which is the harmonic mean of the precision and sensitivity, for the detection of deletions, duplications, insertions, inversions, and translocations of five structural variant (SV) length categories: A (50 - 300bp), B (0.3 - 5kb), C (5 - 50kb), D (50 - 250kb), E (0.25 - 1Mb) using the best combination of SV callers (for details see Material & Methods) based on 25x and 65x Illumina short-read sequencing as well as based on 14x and 25x linked-read sequencing coverage.

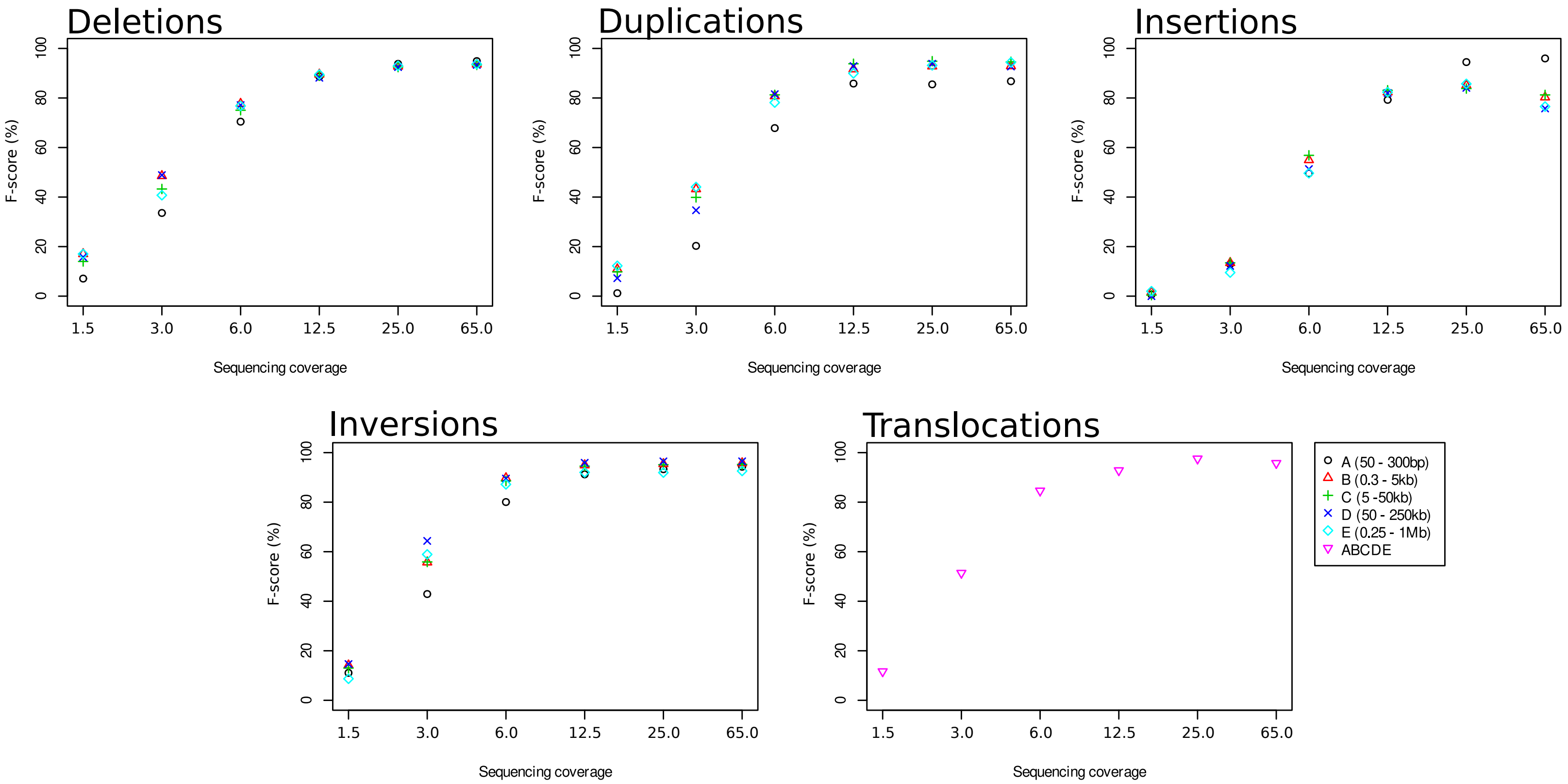

Fig. S2: F1-score, which is the harmonic mean of the precision and sensitivity, for the detection of deletions, duplications, insertions, inversions, and translocations of six sequencing coverages (1.5x, 3.0x, 6.0x, 12.5x, 25.0x, and 65.0x) using the best combination of SV callers (for details see Material & Methods).

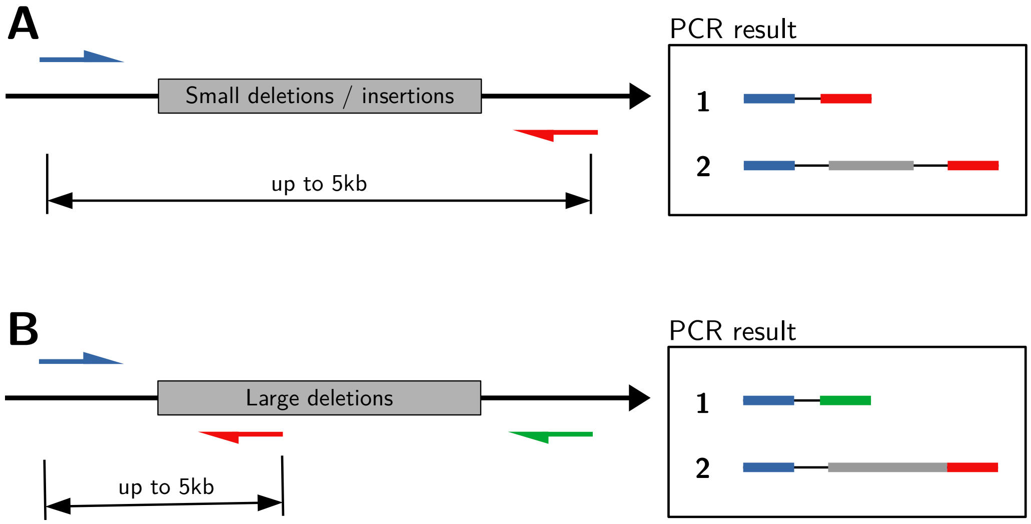

Fig. S3: Graphical illustration of the primer design strategy created to validate structural variant (SV) predictions in the reference genome Morex and Unumli-Arpa. The primer design strategy had to be adjusted depending on the size of the SV. Smaller deletions (A) and insertions (up to ∼5kb) were validated with a pair of two primers (blue/red arrow) flanking the SV (gray box). Larger deletions (B) were validated either by primer 1 (blue) and primer 2 (red) in case of presence or by primer 1 (blue) and primer 3 (green) in case of absence. The predicted PCR results, the absence (1) and presence (2) of the SV sequence in the PCR fragment, are shown on the right.

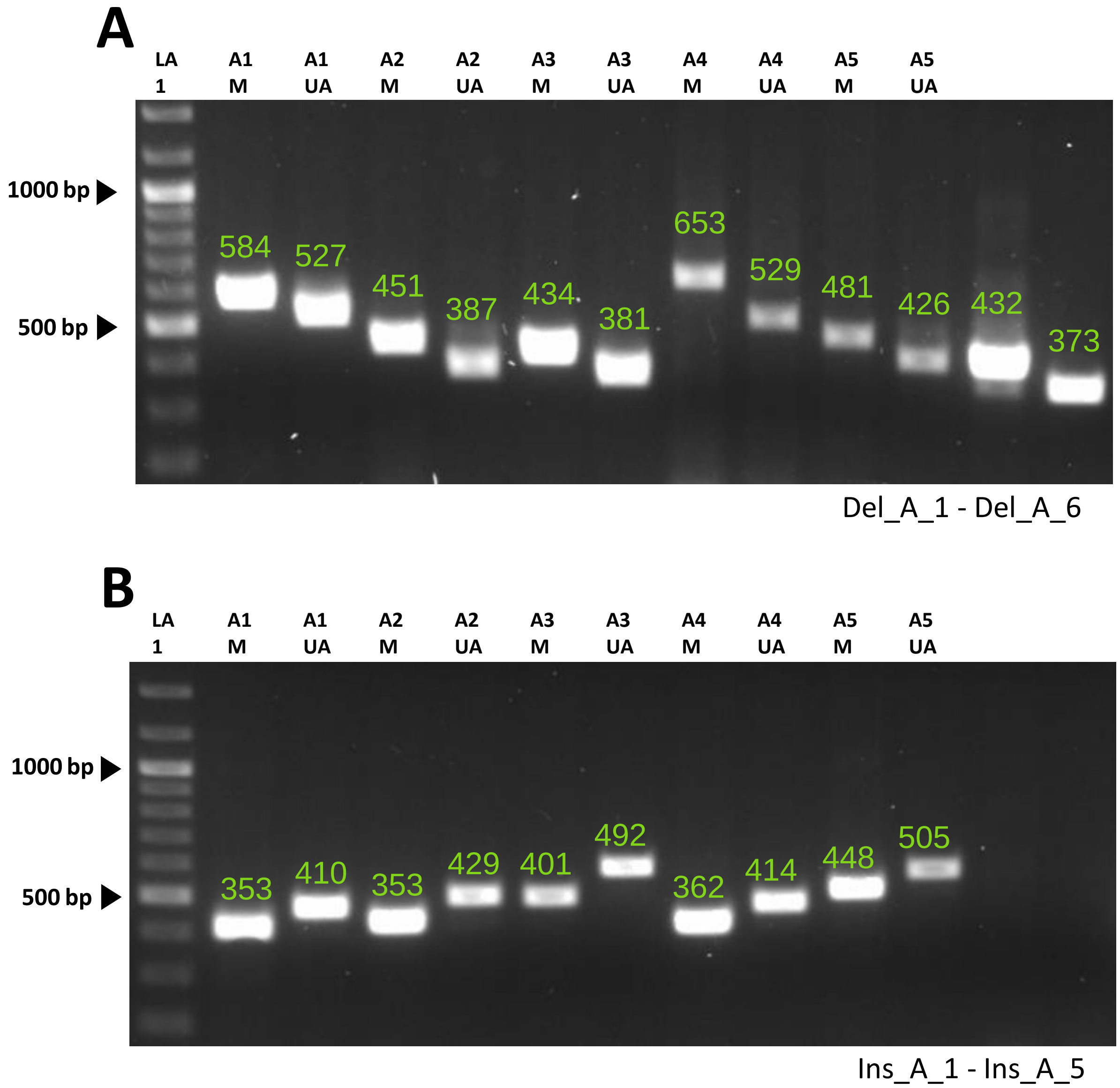

Fig. S4: PCR validation results for small structural variants (SV) as documented after the gel electrophoresis. PCR amplified fragments are shown separated by size for the reference genotype Morex (M) and the genotype Unumli-Arpa (UA). Predicted fragment size based on the SV predictions are illustrated by numbers. The numbers are colored based on the validation success. Fragment size agreement between PCR and prediction (green) or disagreement (red). Results are shown for six small deletions (A) and six small insertions (B) of the SV length category A (50 - 300bp). DNA ladder used: GeneRuler 100bp Plus, Thermo Fisher (LA 1).

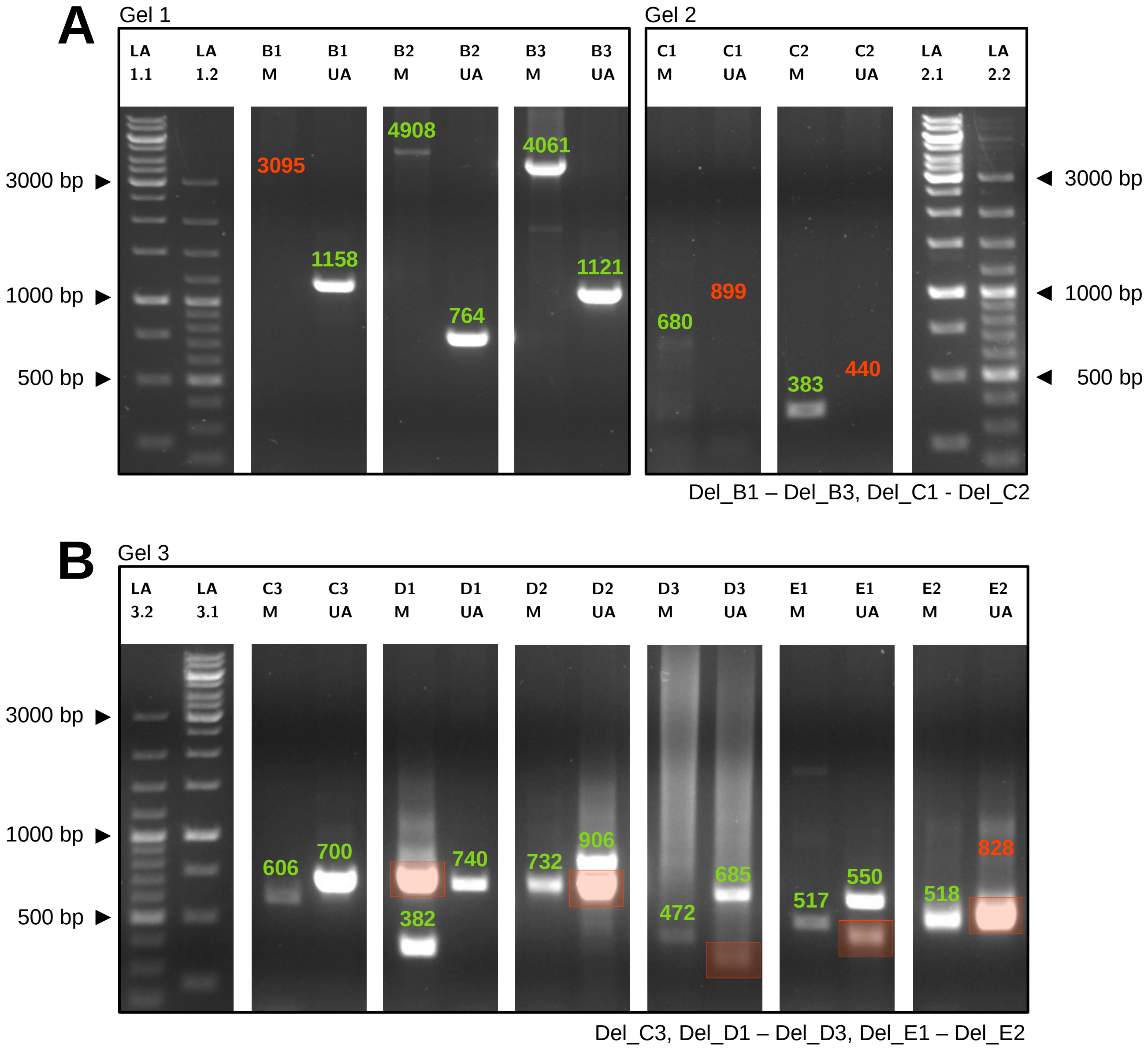

Fig. S5: PCR validation results for large structural variants (SV) as documented after the gel electrophoresis. PCR amplified fragments are shown separated by size for the reference genotype Morex (M) and the genotype Unumli-Arpa (UA). Predicted fragment size based on the SV predictions are illustrated by numbers. The numbers are colored based on the validation success. Fragment size agreement between PCR and prediction (green) or disagreement (red). Additional not predicted fragments are marked by a red box. Results are shown for six deletions of the SV length category B (0.3 - 5kb) (A) and 8 deletions of the SV length category C (5 - 50kb), D (50 - 250kb), and E (0.25 - 1Mb) (B). DNA ladder used: GeneRuler 100bp Plus (LA 1) and GeneRuler 1kb, Thermo Fisher (LA 2).

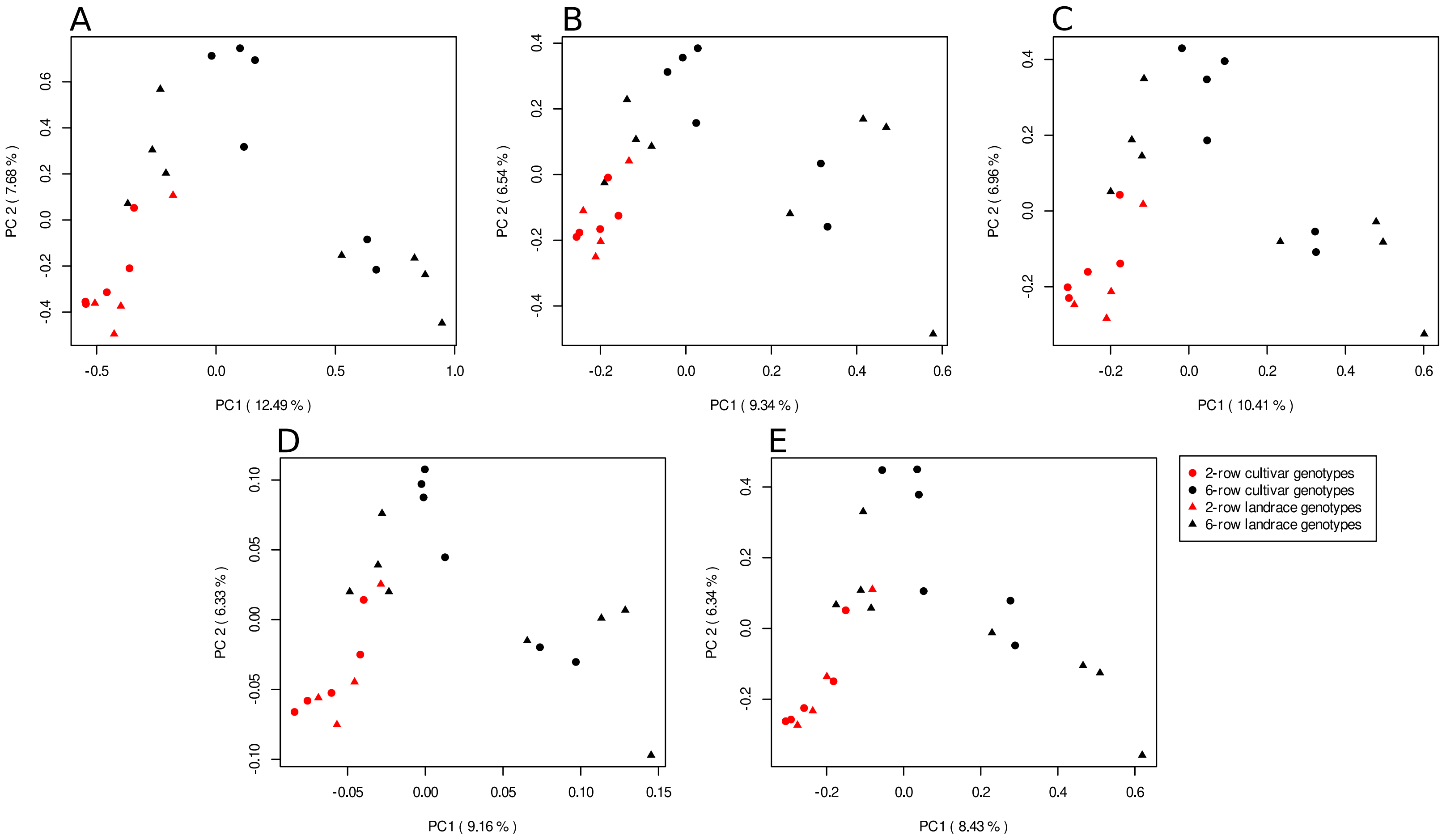

Fig. S6: Principal component analyses of the barley inbred lines considered in our study based on deletions (A), duplicatons (B), insertions (C), inversions (D), and translocations (E). PC 1 and PC 2 are the first and second principal component, respectively, and number in parentheses refer to the proportion of variance explained by the principal components. Symbols identify landrace and cultivar inbreds and colors their row number.

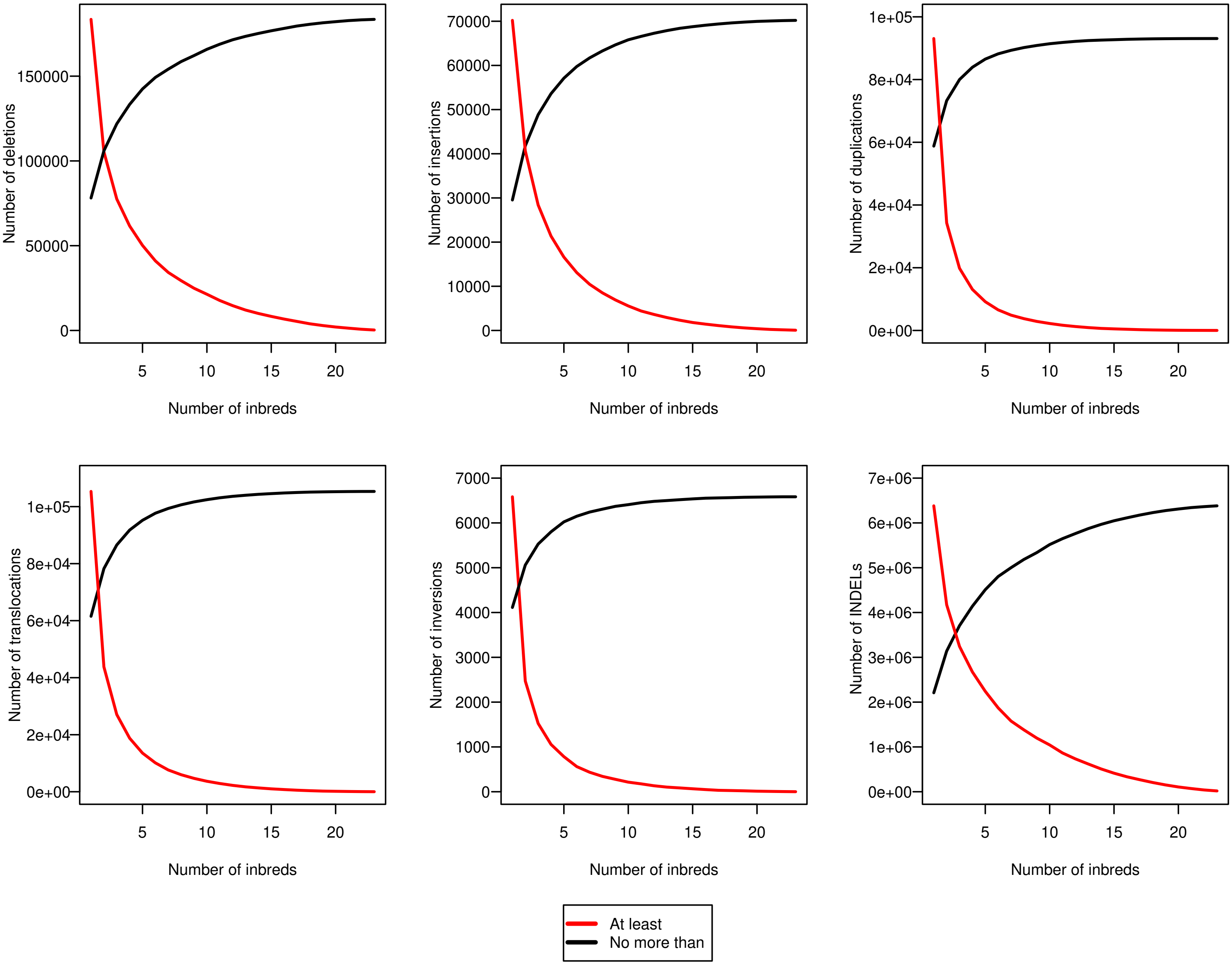

Fig. S7: Number of structural variant (SV) clusters for the different types of SV which were detected in at least (red) or no more than (black) the given number of inbreds.

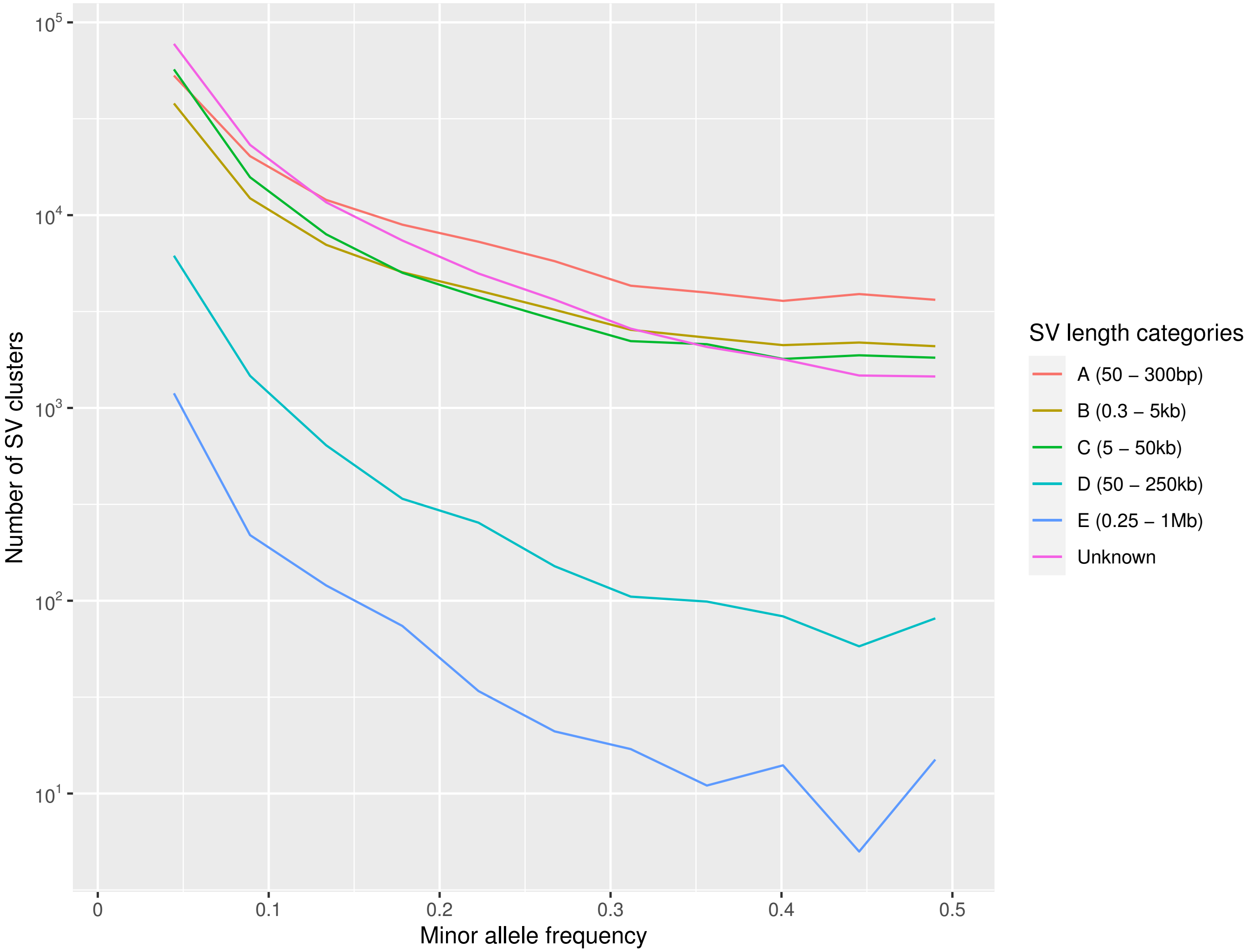

Fig. S8: Detection frequencies of structural variant (SV) clusters of different length categories across the 23 barley inbreds.

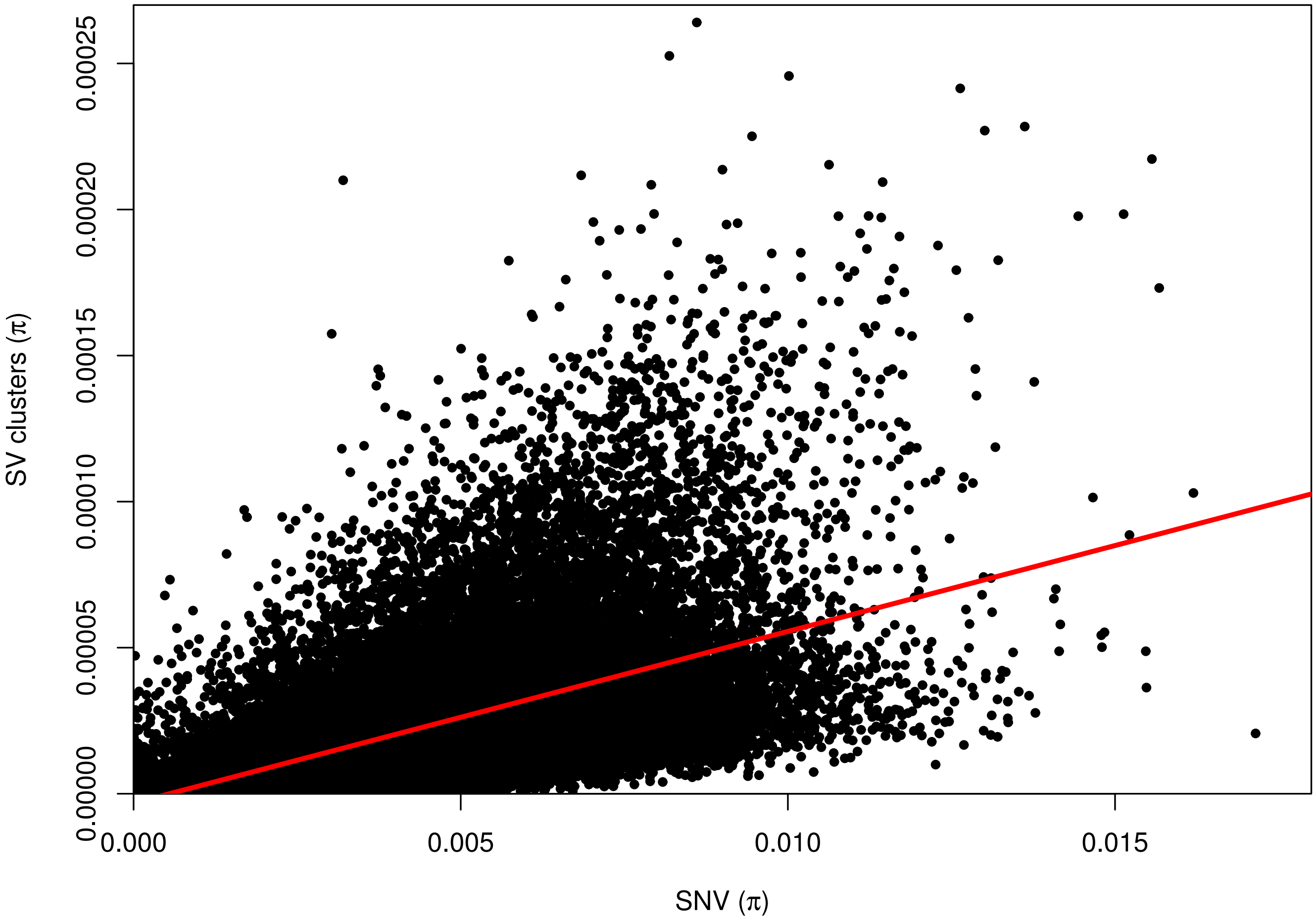

Fig. S9: Average genetic diversity (π) of SNV and SV clusters across 100kb windows of the genome. The red line indicates the correlation.

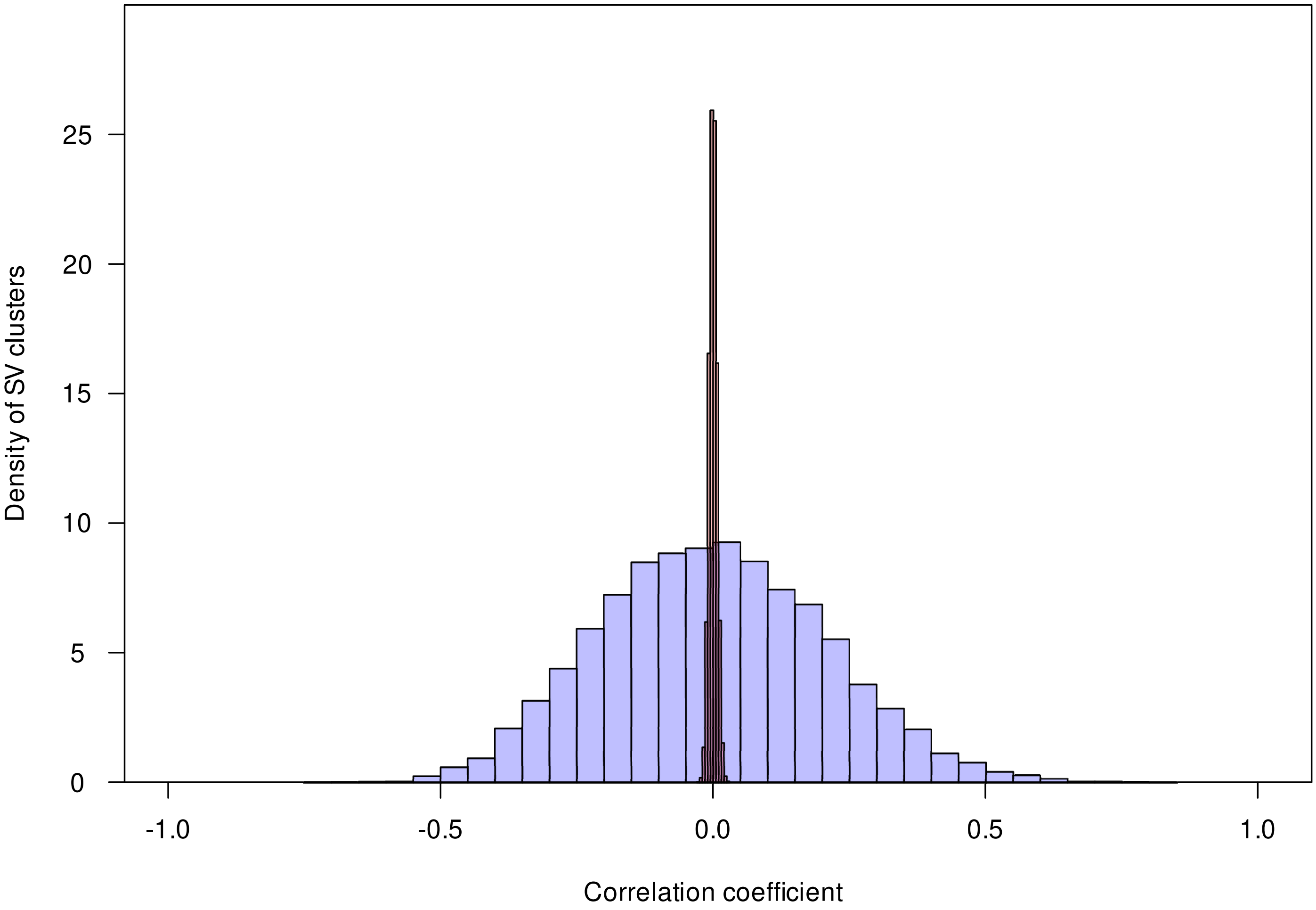

Fig. S10: Distribution of correlation coefficients of presence/absence pattern of all structural variant (SV) clusters (deletions, insertions, duplications, inversions) with minor allele frequency > 0.15 and the loadings of principal component 1 (19.7%) from a principal component analysis of gene expression data. The blue histogram shows the distribution for the detected SV clusters whereas the red histogram shows the distribution for random SV clusters with identical allele frequency.

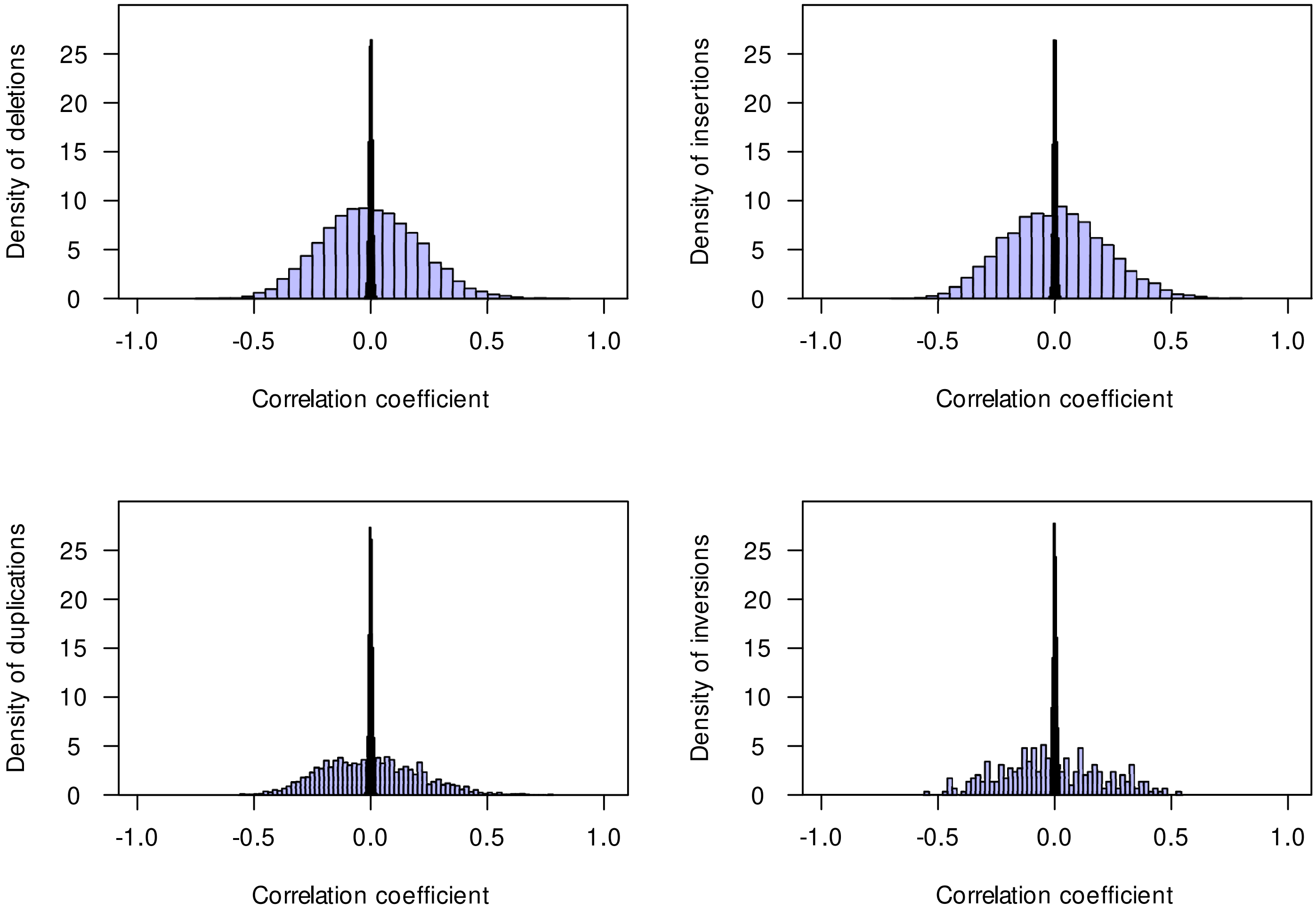

Fig. S11: Distribution of correlation coefficients of presence/absence pattern of deletions, insertions, duplications, and inversions with minor allele frequency > 0.15 and the loadings of principal component 1 (19.7 %) from a principal component analysis of gene expression data. The blue histogram shows the distribution for the detected SV clusters whereas the red histogram shows the distribution for random SV clusters with identical allele frequency.

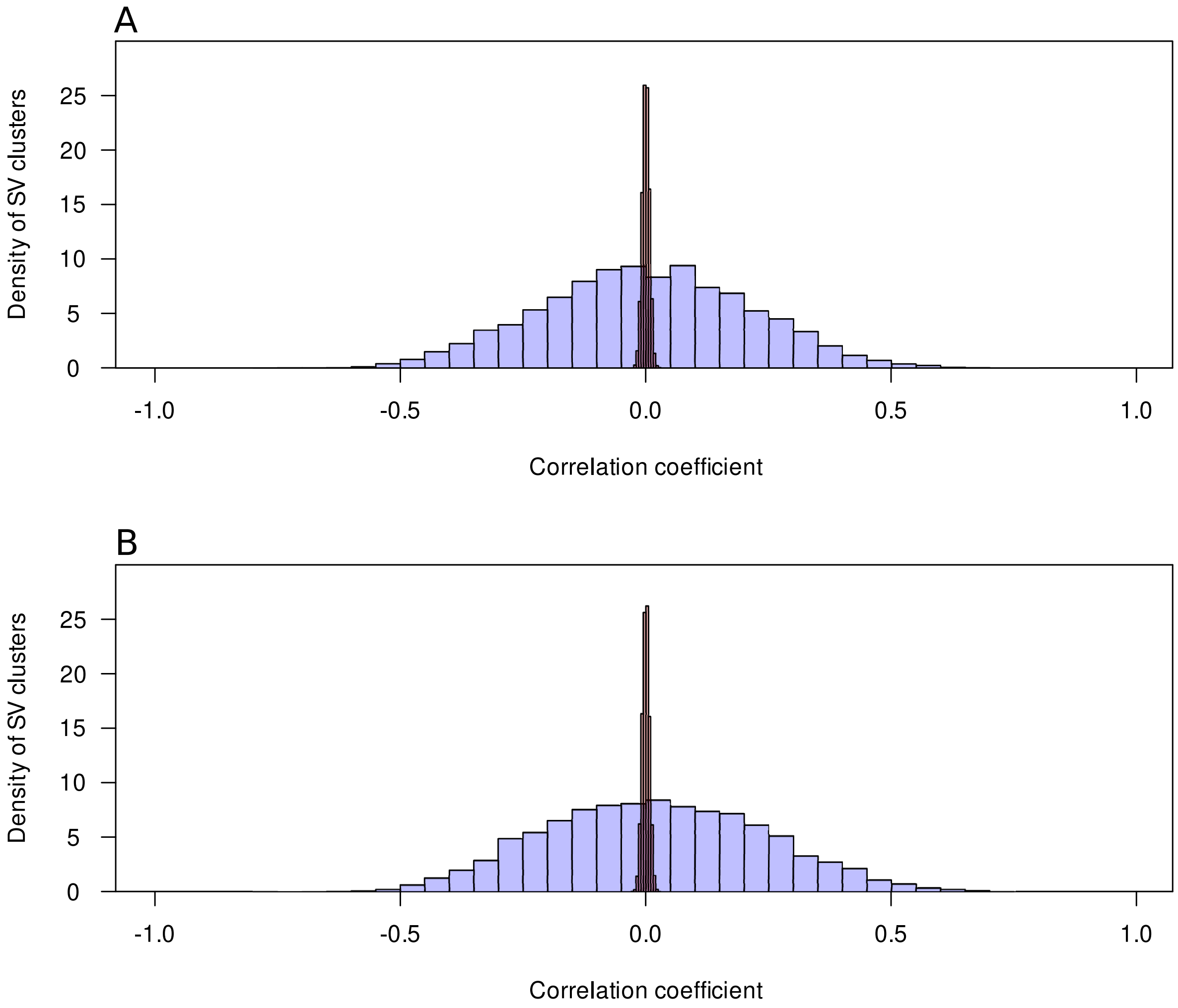

Fig. S12: Distribution of correlation coefficients of presence/absence pattern of SV clusters with minor allele frequency > 0.15 and the loadings of principal component 2 (8.2 %) (A), and 3 (7.1 %) (B) from a principal component analysis of gene expression data. The blue histogram shows the distribution for the detected SV clusters whereas the red histogram shows the distribution for random SV clusters with identical allele frequency.

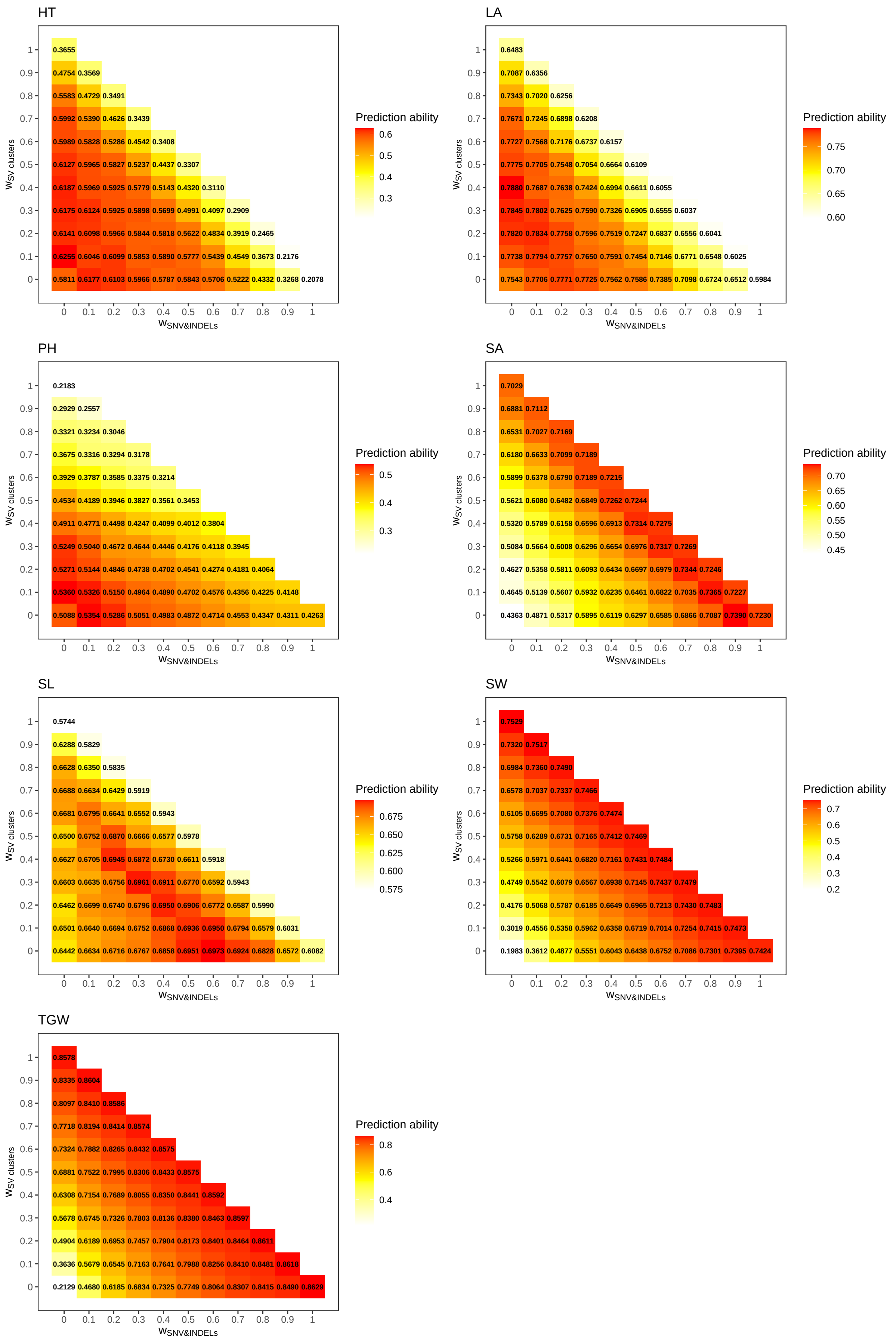

Fig. S13: Prediction ability for the seven phenotypic traits heading time (HT), leaf angle (LA), plant height (PH), seed area (SA), seed length (SL), seed width (SW), and thousand grain weight (TGW) from 23 inbreds for 66 combinations of the joined weighted matrices which differ in the weights of three predictors single nucleotide variants (SNV) and small insertions and deletions (2 - 49bp, INDELs, SNV&INDELs, x-axis), structural variant (SV) clusters (y-axis), and gene expression. Plotted values represent medians across 200 cross-validation runs.
